# B-Cell Lymphoma of the Mandible: A Case Report

**DOI:** 10.4137/cmo.s366

**Published:** 2008-06-11

**Authors:** Ali Adouani, Jed Bouguila, Yassine Jeblaoui, Mehdi Ben Aicha, Mouhamed Ali Abdelali, Mouna Hellali, Karima Zitouni, Landolsi Amani, Zairi Issam

**Affiliations:** Departement of Maxillofacial and Plastic Surgery. Charles Nicolle Hospital. Tunis-Tunisia (Head of the department: Ali Adouani)

**Keywords:** B-cell lymphoma, non-Hodgkin’s lymphoma, mandible, intraosseous lymphoma

## Abstract

**Introduction:**

The mandible is an infrequent localisation of primary osseous non-Hodgkin’s lymphomas. Few cases of mandibular non-Hodgkin’s lymphomas (NHL) have been reported.

**Case report:**

A rare condition of primary malignant non-Hodgkin’s lymphoma of the mandible in 53-year-old man, was reported at the Department of Maxillofacial and Plastic Surgery in Charles Nicolle Hospital (Tunis, Tunisia).

Histologic and Immunohistochemical (IHC) examination Confirmed a B-Cell lymphoma.

**Discussion:**

The purpose of this report is to describe this rare case of NHL of the mandible, explore the diagnosis and workup, and discuss treatment strategies.

In this localisation, neither the clinical features nor the radiologic appearances are often pathognomonic.

**Conclusion:**

Particular care must be taken to consider lymphoma in the differential diagnosis because this uncommon lesion can pose significant diagnostic problems and is frequently misdiagnosed.

## Introduction

Non-Hodgkin’s lymphoma (NHL) arises primarily within the lymph nodes, but approximately 24% affect extranodal locations.^1^,^2^ The most frequent extranodal sites include the gastrointestinal tract, skin, bones, and Waldeyer’s ring.^1^,^3^ NHL of bone is rare, representing only 5% of all extranodal lymphomas.^3^

The mandible accounts for only 0.6% of isolated malignant non-Hodgkin’s lymphomas.^2^ This uncommon localisation can pose significant diagnostic problems and is frequently misdiagnosed.^4^,^5^

We present a rare case of primary lymphoma of the mandible. To our knowledge, there are only a few reports on this condition.

## Case Report

A 53-year-old male with a past history of vague pain along his left mandible was referred to the department of Maxillofacial and Plastic Surgery at Charles Nicolle Hospital.

Six months previously he had undergone the extraction of the sixth lower left tooth.

He then began noting pain in the vicinity of the previous extraction site and left lower lip numbness developed.

**Figure 1:**
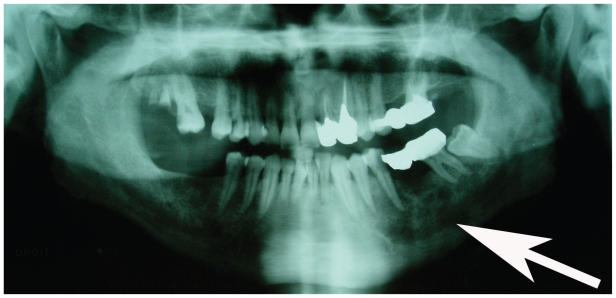
Panoramic radiograph showing a diffuse radiolucent lesion in lower left molar region.

The panoramic radiograph showed a large radiolucent lesion with irregular margins in the left part of the mandible (Fig. 1).

This was thought to be “osteomyelitis” or an”abscess” and he was treated with a course of antibiotics.

Then, the patient presented a gradually swelling on the left mandibular in molar area, with paresthesia of the ipsilateral lower lip.

Extraoral examination showed a left mandibular body swelling, that measured 3*3 cm, expanding both laterally and inferiorly with no lymphadenopathy.

The intraoral examination showed the presence of an expansive lesion of the mandible with normal appearing overlying mucosa. It was tender and painful.

At the time of presentation, the patient did not report any systemic symptoms and had no fever, weight loss, or dysphagia.

**Figure 2:**
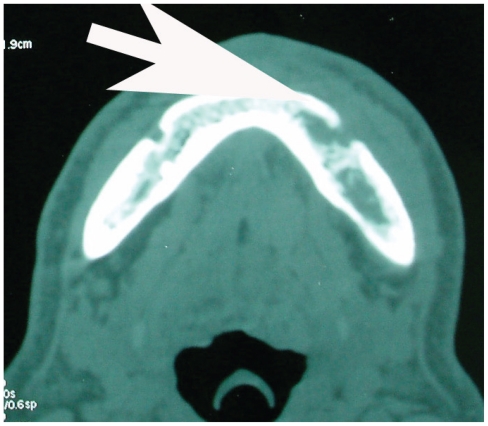
Computed tomagraphy showing a wide osteolytic area in the left part of the mandible.

**Figure 3:**
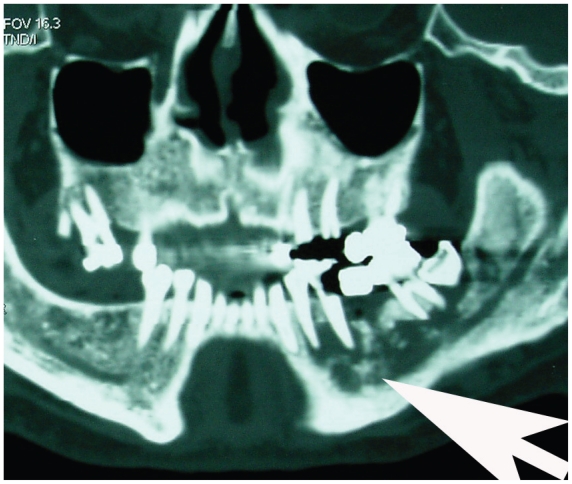
Computed tomography showing a wide osteolytic area in the left part of the mandible.

Computed tomography (CT) of the head and neck performed with i.v. ioded medium showed a wide osteolytic area in the left part of the mandible (Figs. 2, 3); no presence of latero-cervical lymph node lesions was noted.

**Figure 4:**
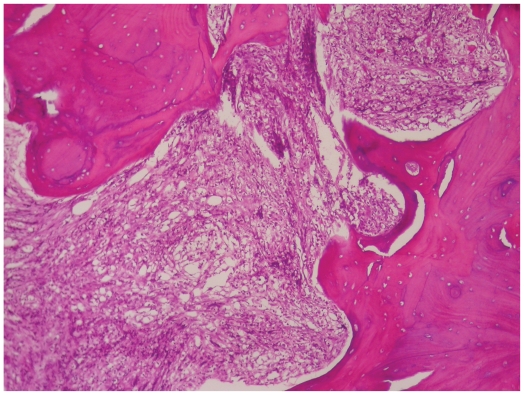
Photomicrograph of the lymphoma showing bony trabeculae infiltrated by lmphoid population with a diffuse growth pattern (H-E).

Incisional biopsy was done and showed dense infiltration of cells with small round hyper chromatic nucleolus and scant cytoplasm resembled lymphocyte (Fig. 4).

**Figure 5:**
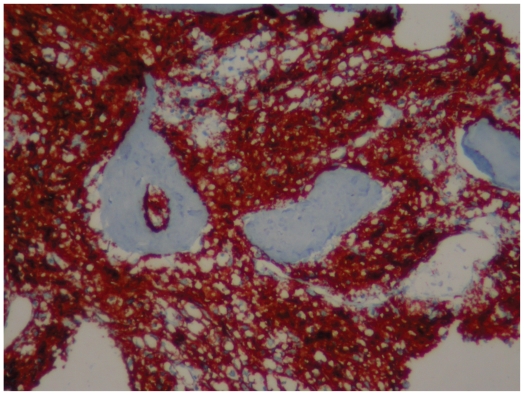
Photomicrograph of immunohistochemical stain shows sheets of large mononuclear cells positive for CD20.

Immunohistochemical (IHC) examination revealed CD20 and PanB positive cells (Fig. 5). This finding confirmed a B-Cell origin for this monomorphic proliferation.

The total body CT scan with i.v.ioded medium and bone marrow aspiration biopsy confirmed the mandible as the only localisation of the pathology. He therefore was categorized as stage IAE, primary mandibular NHL.

The patient refuses all treatment by radio and chemotherapy. He has remained free of disease 10 months after surgery. A follow-up panoramic radiograph showed new bone formation after successful treatment.

## Discussion

The lymphomas are a group of neoplasms arising in the reticuloendothelial and lymphatic system.^4^,^6^ They are divided into 2 major categories: non-Hodgkin’s lymphoma (NHL) and Hodgkin’s disease (HD). Although these diseases usually present with lymphadenopathy or splenomegaly, extranodal sites, including bone tissues, can be involved with disease.^4^,^7^,^8^

Malignant non-Hodgkin’s lymphomas may involve the medullar part of the bone during their evolution but only rarely arise primarily there.^9^,^10^ We present a rare case of primary lymphoma of the mandible. To our knowledge, there are only a few reports on this condition. Most such lymphomas have been shown to be predominantly of B-cell lineage.^11^

Primary mandibular involvement makes up about 0.6% of all malignant non-Hodgkin’s lymphomas,^5^ 5% of all NHL of bone and accounts for 8% all mandibular tumors.^3^,^8^

The most frequent site of occurrence in the mandible is the body.^7^,^8^,^12^

Owing to their low frequency and to their not specific symptoms, primary malignant non-Hodgkin’s lymphomas of the mandible are often misdiagnosed.^1^,^9^,^11^ These signs may lead to an incorrect diagnosis because of the resemblance to other common dental disease.^1^,^12^ the clinical presentation mirrors that of an odontogenic process or localized osteomyelitis.^3^,^13^ Lesions are sometimes thought to be inflammatory or infectious, therefore delaying diagnosis as was the case seen with the patient presented here.^1^,^3^,^7^

The clinical presentation of mandibular NHL usually involves localized bone swelling, teeth mobility,^2^,^14^ mass in an extraction socket, pain, pathologic fracture and often anesthesia or paresthesia along the distribution of the inferior alveolar nerve.^3^,^11^

In this patient, Lymphoma was presented as mandibular intraosseous lesion. Paresthesia in mental region indicate compression or infiltration of mandibular nervous tissue.

Despite extensive local destruction, systemic manifestations of malignant disease, such as weight loss, fever, and malaise, were very minimal.^7^

This patient’s clinical presentation of a painful mass was consistent with reports of localized pain present at presentation. This mandibular swelling was initially thought to be due to an inflammatory odontogenic/periodontal process and was treated as such with antibiotic and dental extraction.

In the head and neck region, lymphoma should be considered when there is unexplained dental pain, numbness, tooth mobility, swelling, ulceration, a mass in an extraction socket, or any ill-defined lytic osseous changes.^1^

Histopathologic evaluation, together with immunophenotypic and cytogenetic studies, elucidate the histologic type.^3^ Immunohistochemistry plays an important role in distinguishing cell types and differential diagnosis;^1^,^13^ a panel of monoclonal antibodies are used: CD20 (selective marker that recognizes a subpopulation of B-cells); CD10 (marker for follicular center B-cells and granulocytes), CD3 (marker for T-cells and NK cells), CD30 (marker for activated T- and B-cells), ALK-1 (marker for anaplastic large cells) and Ki 67 (proliferation marker).^1^,^12^ For this case, the diagnosis was based on limited immunohistochemistry (CD20 and PanB).

Diffuse large B-cell lymphoma (formerly known as diffuse histiocytic lymphoma) is the most common subtype of NHL, including primary mandibular NHL.^3^ In NHL of bone the cell architecture and histology is indistinguishable from nodal or lymphoid derived lymphomas.^1^,^3^

The work up should assess the extent of disease and allow for accurate staging.

The current staging procedures for extranodal oral lymphoma include a panoramic radiograph, abdominal and chest CT scan, bone scans, routine laboratory tests (hemogram, urine analysis, complete blood cell counts, lactate dehydrogenase and erythrocyte sedimentation rate), and bone marrow aspiration biopsy.^1^,^3^,^7^,^13^

There are no radiographic pathognomonic findings.^3^,^9^,^12^ Radiographic features do not have specific characteristics, showing an osteolytic area. Often small areas of calcification can be seen in these masses.^7^ Panoramic radiograph and CT scan are used to demonstrate local extension of non-Hodgkin’s lymphoma, whereas total body CT scan with i.v. ioded medium and/or osseous scintiscan are used to exclude other sites and to stage the pathology.^9^

To be classified as a primary NHL of bone there must be no evidence of visceral or lymphatic involvement and no distant metastases for at least 6 months following diagnosis.^3^ The most widely used system for staging lymphomas is the Ann Arbor classification, initially introduced for Hodgkin’s lymphoma and later adopted to classify non-Hodgkin’s lymphomas.^5^,^11^,^15^ Stage I comprises lymphomas localized and confined to one side of the diaphragm with a single lesion; Stage IE indicates a single extralymphatic organ or site involved.^1^,^16^

Stage I NHL has a 5-year survival rate of 70% and the median survival time for IE is 10 years.^2^,^3^ The 5-year survival rate for stage IE NHL of the maxillo-mandibular region is reported to be approximately 50%.^2^,^3^,^10^

Treatment for primary lymphoma of the mandible typically consists of a combination of chemotherapy and radiotherapy^3^,^10^,^15^ and surgical approach is limited to obtaining a specimen representative of the lesion and sufficient for a complete histological examination.^5^,^9^

The role of surgery is for biopsy purposes and for the control of persistent or recurrent local disease.^3^

For this patient the follow-up was extremely short; the treatment consisted of only surgical excision, he showed complete remission with the disappearance of all clinical evidence of disease and the normalization radiologic abnormalities related to the disease. In spite of this clinical remission, a complement of radio and chemotherapy is discussed to improve prognosis but the patient refused that treatment.

## Conclusion

Lymphomas are the most frequent nonepithelial malignant tumors in the oral cavity and maxillofacial region and represent the third most common group of malignant lesions in this site, following squamous cell carcinoma and salivary gland neoplasms.^1^,^11^

We present a case of isolated mandibular NHL, stage IE. This report discusses this rare malignancy, including clinical presentation, histopathologic features, immunologic profile, treatment, and prognosis.

## References

[1] Angiero F, Stefani M, Crippa R (2006). Primary non-Hodgkin’s lymphoma of the mandibular gingiva with maxillary gingival recurrence. Oral Oncology EXTRA.

[2] Bachaud JM, Coppin D, Douchez J (1992). Les lymphomes malins primitifs de la mandibule. Etude de 3 cas et revue de la litterature. Rev Stomatol Chir Maxillofac.

[3] Steinbacher DM, Dolan RW (2006). Isolated non-Hodgkin’s lymphoma of the mandible. Oral Oncology EXTRA.

[4] Ugar DA, Turker M, Memis L (1995). Primary Lymphoma of the Mandible: Report of a Case. J Oral Maxillofac Surg.

[5] Gusenbauer AW, Katsikeris NF, Brown A (1990). Primary lymphoma of the mandible: report of a case. J Oral Maxillofac Surg.

[6] Mojaver YN, Sahebjamie M, Tirgary F (2005). Enlargement of mandibular canal with tongue paresthesia caused by extranodal B-cell Lymphoma: A case report. Oral Oncology EXTRA.

[7] Kirita T, Ohgi K, Shimooka H (2000). Primary non-Hodgkin’s lymphoma of the mandible treated with radiotherapy, chemotherapy, and autologous peripheral blood stem cell transplantation. Oral Surg Oral Med Oral Pathol Oral Radiol Endod.

[8] Piatelli A, Croce A, Tete S, Artese L (1997). Primary non-Hodgkin’s lymphoma of the mandible: A case report. J Oral Maxillofac Surg.

[9] Longo F, De Maria G, Esposito P, Califano L (2004). Primary non-Hodgkin’s lymphoma of the mandible Report of a case. Int J Oral Maxillofac Surg.

[10] Barbieri E, Cammelli S, Mauro F (2004). Primary non-hodgkin’s lymphoma of the bone: treatment and analysis of prognostic factors for stage I and stage II. Int J Radiation Oncology Biol Phys.

[11] Kolokotronis A, Konstantinou N, Christakis I (2005). Localized B-cell non-Hodgkin’s lymphoma of oral cavity and maxillofacial region: A clinical study. Oral Surg Oral Med Oral Pathol Oral Radiol Endod.

[12] Cox DP, Treseler P, Dong R, Jordan RCK (2007). Rare oral cavity presentation of a B-cell lymphoblastic lymphoma. A case report and review of the literature. Oral Surg Oral Med Oral Pathol Oral Radiol Endod.

[13] Kawasaki G, Nakai M, Mizuno A (1997). Malignant lymphoma of the mandible Report of a case. Oral Surg Oral Med Oral Pathol Oral Radiol Endod.

[14] Leong IT, Fernandes B, Mock D (2001). Epstein-Barr virus detection in non-Hodgkin’s lymphoma of the oral cavity: an immunocytochemical and in situ hybridization study. Oral Surg Oal Med Oral Pathol Oral Radiol Endod.

[15] Yamada T, Kitagawa Y, Ogasawara T (2000). Enlargement of mandibular canal without hypesthesia caused by extranodal non-Hodgkin’s lymphoma. Oral Surg Oral Med Oral Pathol Oral Radiol Endod.

[16] Ishimaru T, Hayatsu Y, Ueyama Y, hinozaki F (2005). Hodgkin’s Lymphoma of the Mandibular Condyle: Report of a Case. J Oral Maxillofac Surg.

